# The Response of Water Dynamics to Long-Term High Vapor Pressure Deficit Is Mediated by Anatomical Adaptations in Plants

**DOI:** 10.3389/fpls.2020.00758

**Published:** 2020-06-05

**Authors:** Qingjie Du, Xiaocong Jiao, Xiaoming Song, Jiayu Zhang, Ping Bai, Juping Ding, Jianming Li

**Affiliations:** ^1^College of Horticulture, Northwest A&F University, Yangling, China; ^2^College of Horticulture, Henan Agricultural University, Zhengzhou, China

**Keywords:** anatomical acclimations, hydraulics, stomatal conductance, transpiration, vapor pressure deficit

## Abstract

Vapor pressure deficit (VPD) is the driver of water movement in plants. However, little is known about how anatomical adaptations determine the acclimation of plant water dynamics to elevated VPD, especially at the whole plant level. Here, we examined the responses of transpiration, stomatal conductance (g_s_), hydraulic partitioning, and anatomical traits in two tomato cultivars (Jinpeng and Zhongza) to long-term high (2.2–2.6 kPa) and low (1.1–1.5 kPa) VPD. Compared to plants growing under low VPD, no variation in g_s_ was found for Jinpeng under high VPD conditions; however, high VPD induced an increase in whole plant hydraulic conductance (K_plant_), which was responsible for the maintenance of high transpiration. In contrast, transpiration was not influenced by high VPD in Zhongza, which was primarily attributed to a coordinated decline in g_s_ and K_plant_. The changes in g_s_ were closely related to stomatal density and size. Furthermore, high VPD altered hydraulic partitioning among the leaf, stem, and root for both cultivars via adjustments in anatomy. The increase in lumen area of vessels in veins and large roots in Jinpeng under high VPD conditions improved water transport efficiency in the leaf and root, thus resulting in a high K_plant_. However, the decreased K_plant_ for Zhongza under high VPD was the result of a decline of water transport efficiency in the leaf that was caused by a reduction in vein density. Overall, we concluded that the tradeoff in anatomical acclimations among plant tissues results in different water relations in plants under high VPD conditions.

## Introduction

The process of water movement through soil–plant–atmosphere continuum (SPAC) is driven by atmospheric evaporative demand which can be expressed as vapor pressure deficit (VPD). Although the optimal VPD for most greenhouse crops is below 1.5 kPa (Shamshiri et al., 2016), high VPD (>2 kPa) is currently observed in greenhouses, especially during summer (Lu et al., 2015; Zhang et al., 2018). For plants grown under high VPD conditions, a central question is how they regulate transpiration (Carins Murphy et al., 2014; Allen et al., 2015; Grossiord et al., 2017). In a plant, water absorbed through the roots is transported to leaves through the xylem, finally lost via stomata by diffusion. Hence, the regulation of transpiration may occur at whole plant levels. However, the responses of physiological and anatomical traits that could influence transpiration remain largely unknown at whole plant levels during high VPD condition.

Most of the water loss by a plant occurs through stomatal apertures (Macková et al., 2013). Moreover, cuticular pathway is found to be important in regulating water loss when stomatal closure takes place (Fanourakis et al., 2013, 2019). Under steady state conditions and in the vapor phase, the transpiration rate (E) is defined mathematically as a function of stomatal conductance (g_s_) and VPD. Although the regulation of transpiration depends on the response of g_s_ to VPD during the vapor phase, the efficiency of the hydraulic system determines the amount of liquid water lost to evaporation for any given soil water condition. Using an analogy of Ohm’s law, E can be expressed as the product of hydraulic conductance and water potential gradient in liquid flux. Many studies have proposed a hydraulic feedback loop to interpret the dynamic link between the liquid and gas phases (Buckley, 2005; Domec et al., 2009; Simonin et al., 2015). Thus, a coordination may exist between g_s_ and whole plant hydraulic conductance (K_plant_) with respect to water transport across the soil–plant–atmosphere continuum.

To deal with long-term environmental fluctuation, plants have evolved high plasticity in carbon allocation (Freschet et al., 2018). The changes in carbon investment generally trigger adjustments in anatomical traits involved in plant water dynamics, which occur at multiple places in the plant including stomatal and xylem tissues (Sperry et al., 1998; de Boer et al., 2016; Dewar et al., 2018). Previous studies demonstrates stomatal density decreased to prevent excessive water loss under high VPD in tomato (Lu et al., 2015; Du et al., 2019), rose (Fanourakis et al., 2012), and fava bean (Aliniaeifard et al., 2014). However, few stomata mean a reduction in the maximum potential carbon acquisition. Alternatively, plants resort to an efficient hydraulic system to withstand excessive evaporative demand. Despite a high carbon investment to xylem, these adjustments contribute to maintain carbon acquirement. Thus, a tradeoff between water loss and carbon acquirement would exist during the acclimation process of plants to high VPD. Additionally, the hydraulic system in plants shows a strong hydraulic segmentation (Cruiziat et al., 2002; Sperry and Love, 2015). Although the dynamics of hydraulic conductance of leaves, stems, and roots have been well-documented in response to soil water deficit (Domec et al., 2009; Torres-Ruiz et al., 2015), the adjustment of hydraulic structure to long-term high VPD has been poorly investigated. Therefore, systematic knowledge about acclimation at the whole plant level is critical to determining the responses of plants to high VPD and is necessary to understand the tradeoff between carbon investment in regulating water dynamics and carbon acquirement.

Tomato (*Solanum lycopersicum* L.) is one of the most important agricultural plants in the world. High VPD induces contrasting responses in plant water status among tomato cultivars (Zhang et al., 2018; Du et al., 2019). In the present study, two tomato cultivars, Jingpeng and Zhongza, were selected on the basis that they exhibit different responses to altered VPD (Du et al., 2019). The responses of water dynamics and anatomical traits were measured on these two cultivars after exposure to long-term high and low VPD. We hypothesized that (1) for plants with high water loss under high VPD, K_plant_ would increase with unaffected g_s_; (2) for plants with relatively low water loss under high VPD, K_plant_ and g_s_ would synchronously decline; and (3) acclimation in terms of water dynamics is related to anatomical changes at multiple places in plants.

## Materials and Methods

### Plant Material and Growth Conditions

Seeds of Jinpeng and Zhongza were germinated and grown in plastic pots [15 cm × 10 cm (diameter × height); 1 plant/pot] containing a mixed peat-perlite substrate. The seedlings were kept in a walk-in growth chamber. The light in the chambers was given daily for 14 h at a photon flux density of 300 μmol m^–2^ s^–1^. The temperature was 28–30°C day/19–20°C night. Relative humidity was regulated between 64–70% day/77–82% night using an ultrasonic humidifier (KAJ-9.0B, Kawasima Appliance Co., Ltd., Changzhou, China) and dehumidifier (DH-702B, Chuanjing Electric Co., Ltd., Hangzhou, China). Consequently, the VPD was 1.1–1.5 kPa day/0.4–0.5 kPa night. After 5 weeks, 30 of the healthiest plants were divided into two random groups of 15 for each cultivar. For low VPD treatment, the plants were kept on previous humidity conditions. A high VPD was performed by setting 36–42% relative humidity during the day (VPD 2.2–2.6 kPa). Plants were grown for 30 d and kept well-watered during the entire growth period. New fully expanded leaves were used for measurements.

### Transpiration and Stomatal Conductance

To determine transpiration at the canopy level (E_canopy_) during the photoperiod, pots were covered with plastic film and aluminum foil on the day before measurement. After at least 2 h of acclimation in the photoperiod, five pots were weighed 2 and 8 h after turning on the lights. E_canopy_ was calculated by differences in weight divided by the total leaf area. Canopy stomatal conductance (g_s_-*canopy*) was determined according to a simplified inversion of the Penman–Monteith model (Monteith and Unsworth, 1990):

$$g_{s-c\,a\,n\,o\,p\,y}=\frac{R\,T_{a}\,\rho \,E_{c\,a\,n\,o\,p\,y}}{V\,P\,D},$$

where *R* is the universal gas constant adjusted for water vapor (0.46 m^3^ kPa K^–1^ kg^–1^), *T*_a_ is air temperature (K), and ρ is the density of water (998 kg m^–3^).

Leaf level transpiration (E_leaf_) and stomatal conductance (g_s–leaf_) were measured with a plant porometer (Yaxin-1301, Yaxin Liyi Technology Co., Ltd., Beijing, China). After at least 2 h of acclimation in the photoperiod, five leaves from different plants for each treatment and cultivar were used for measurements. VPD, light, and the temperature of the cuvette were kept at ambient levels.

### Hydraulic Conductance

Hydraulic conductance of the leaf (K_leaf_), stem (K_stem_), and root (K_root_) and K_plant_ were calculated according to Domec et al. (2009). Briefly,

$$K_{p\,l\,a\,n\,t}=\frac{E_{l\,e\,a\,f}}{\Psi_{s\,o\,i\,l}-\Psi_{l\,e\,a\,f}},$$

$$K_{l\,e\,a\,f}=\frac{E_{l\,e\,a\,f}}{\Psi_{s\,t\,e\,m-u\,p}-\Psi_{l\,e\,a\,f}},$$

$$K_{s\,t\,e\,m}=\frac{E_{l\,e\,a\,f}}{\Psi_{s\,t\,e\,m-b\,a\,s\,e}-\Psi_{s\,t\,e\,m-u\,p}},$$

$$\frac{1}{K_{r\,o\,o\,t}}=\frac{1}{K_{p\,l\,a\,n\,t}}-\frac{1}{K_{l\,e\,a\,f}}-\frac{1}{K_{s\,t\,e\,m}},$$

where Ψ_soil_ is the soil water potential measured with a PSYPRO Water Potential System (PSYPRO; Wescor, Inc., Logan, UT, United States), Ψ_leaf_ is the water potential of the leaf used for gas exchange measurement (transpiring leaf), Ψ_stem_-*up* is the stem water potential in the upper crown section, and Ψ_stem–base_ is the stem water potential at the stem base. Ψ_stem_ was estimated from the leaf water potential of a non-transpiring leaf (achieved by covering with plastic film and aluminum foil the night before measurement) (Richter, 1997; Zsögön et al., 2015). The leaf adjacent to the transpiring leaf was used for Ψ_stem_-*up* and the first true leaf was used for Ψ_stem_-*base*. Measurements of Ψ_leaf_, Ψ_stem_-*up*, and Ψ_stem_-_base_ were performed on the same five plants used for gas exchange measurements with a pressure chamber (PMS, Corvallis, OR, United States). After measurement of hydraulic conductance, the plants were used for morphological observation.

### Stomatal Characteristics

Stomatal morphological characteristics were determined using the impression method as described by Xu and Zhou (2008). Briefly, epidermis was smeared with nail varnish in the mid-area between the midrib and lateral margin, avoiding midrib and secondary veins (Fanourakis et al., 2015b). Then, the thin film (approximately 5 mm × 5 mm) was peeled off from the leaf surface and mounted on a glass slide. All stomatal characteristics were measured on both the adaxial and abaxial sides of the leaf. For determining stomatal density (SD), five images per sampling area were taken at a magnification of 200× with a light microscope (BX50, Olympus, Tokyo, Japan). Stomatal area (SA) was measured on at least 20 stomata per sampling area at a magnification of 400× with ImageJ software (National Institutes of Health, Bethesda, MD, United States). SA was defined as the combined area of pore and a pair of guard cells following Savvides et al. (2012).

### Vein and Stem Anatomical Traits

To evaluate leaf vein traits, leaflets were detached from the leaves used for Ψ_leaf_ measurements. Cleared leaflets were scanned to estimate the length of the midrib and secondary vein as well as the leaf area. Veins with an order higher than secondary were measured on 1-cm^2^ leaf pieces from the center of each leaf at a magnification of 40× according to Kono et al. (1982). Briefly, the lamina was boiled in 70% ethanol to remove pigment. After washing with distilled water, samples were transferred to boiling 85% lactic acid for 20 min and then spread out flat on a slide for observation. The vein density was calculated as the ratio of total vein length to the analyzed area.

To assess the xylem composition in veins, segments (0.3 cm in length) were cut from the petiole immediately below the lamina insertion point. This section was selected because it was connected to the midrib and water entering the leaflet would have to pass through this part. The middle of the plant was sampled to estimated xylem composition in the stem. After fixing in a mixture of formaldehyde, acetic acid, and alcohol for 24 h, the sample material was dehydrated in a graded ethanol-xylene series and infiltrated with paraffin. Then 12-μm thick sections were made using a rotary microtome, stained with safranin and fast green, and mounted on slides with a cover slip (Berlyn and Miksche, 1976). The cross-sectional area of the petiole and total number of vessels in the petiole were measured at magnifications of 40× and 100×, respectively. Vessel density was defined as the number of vessels per unit area. The lumen area of vessels in the leaf vein (A_lumen_-*leaf*) and stem (A_lumen_-*stem*), and wall thickness of vessels in the stem (T_w_-*stem*), were estimated at a magnification of 400× with ImageJ (at least three different field-of-view regions). The lumen diameter of each vessel was calculated from its lumen area, assuming a circular shape. The maximum theoretical leaf vein axial hydraulic conductivity (K_leaf_-*max*) was estimated as follows (North et al., 2013):

$$K_{l\,e\,a\,f-m\,a\,x}=\sum_{i}^{N}\frac{\pi \,d_{i}^{4}}{128\,\eta },$$

where N is total number of vessels in the petiole, *d*_*i*_ is the lumen diameter of each vessel, and η is the viscosity of water, further normalized by leaf area (Sack and Frole, 2006).

### Root Morphological Characteristics

After measurement of hydraulic conductance, roots were detached from the plant and carefully washed. The cleaned samples were scanned and analyzed with WinRhizo software (WinRhizo, Regent Ltd., Canada).

### Growth Analyses

Five plants per treatment were selected to measure plant biomass and total leaf area. The aboveground and underground dry biomass of plant was determined after drying to a constant weight at 80°C. The net assimilation rate (NAR) was calculated as follows:

$$N\,A\,R=\frac{W_{2}-W_{1}}{T_{2}-T_{1}}\times \frac{l\,n\,L_{2}-l\,n\,L_{1}}{L_{2}-L_{1}},$$

where *W*_1_ and *W*_2_ are total dry weights of the whole plant at times *T*_1_ and *T*_2_; *L*_1_ and *L*_2_ are total leaf areas of the whole plant at times *T*_1_ and *T*_2_.

### Statistical Analysis

All statistical analyses were performed using SPSS 19.0 (IBM Corp., Armonk, NY, United States). One-way ANOVA was used to test differences between mean values (Duncan’s test). Two-way ANOVA was used to determine the main effects of cultivar, treatment, and their interactions.

## Results

### Transpiration and Stomatal Conductance

**FIGURE 1:**
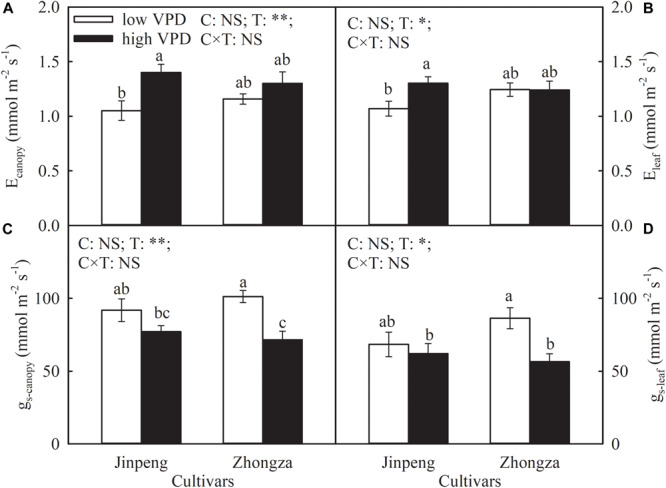
Transpiration rate at the canopy (E_canopy_) **(A)** and leaf level (E_leaf_) **(B)**, stomatal conductance at the canopy (g_s_-*canopy*) **(C)** and leaf level (g_s_-*leaf*) **(D)** for two tomato cultivars, Jinpeng and Zhongza, grown under low (1.1–1.5 kPa) and high (2.2–2.6 kPa) VPD. Data are means ± standard error (*n* = 5 plants). Different letters denote statistically significant differences (Duncan’s test, *P* < 0.05). Two-way ANOVA was used to estimate the effect of cultivar (C), treatment (T), and their interaction (C × T) (^∗∗^*P* < 0.01; ^∗^*P* < 0.05; NS, not significant).

The effects of VPD on E_canopy_ and E_leaf_ were similar and cultivar specific (Figure 1). The E_canopy_ and E_leaf_ of Jinpeng significantly increased under high VPD compared to low VPD. On the contrary, in Zhongza, these measures were both unaffected by VPD treatment. Under high VPD, g_s_-*canopy* and g_s_-*leaf* decreased by 29 and 35%, respectively, in Zhongza relative to low VPD whereas VPD had no influence on either g_s_-*canopy* or g_s_-*leaf* in Jinpeng.

### Stomatal Characteristics

No significant differences in SD or SA for either the adaxial or abaxial sides of the leaf were found between low and high VPD in Jinpeng (Table 1). For Zhongza, high VPD significantly decreased SD on both sides and SA on the adaxial side only. Moreover, the integrated values of SD and SA for the whole leaf also declined under high VPD compared to low VPD in Zhongza.

**TABLE 1:** Stomatal density (SD) and area (SA) for two tomato cultivars, Jinpeng and Zhongza, grown under low (1.1–1.5 kPa) and high (2.2–2.6 kPa) VPD.

**Cultivars**	**Treatments**	**SD_adaxial_ (mm^–2^)**	**SD_abaxial_ (mm^–2^)**	**SD (mm^–2^)**	**SA_adaxial_ (μm^2^)**	**SA_abaxial_ (μm^2^)**	**SA (μm^2^)**
Jinpeng	Low VPD	53.83 ± 3.29 a	132.66 ± 4.67 b	186.49 ± 6.86 ab	277.46 ± 9.92 ab	294.56 ± 11.74 b	286.74 ± 10.74 b
	High VPD	52.91 ± 4.12 a	125.95 ± 2.71 b	178.81 ± 4.45 bc	265.60 ± 8.20 b	300.01 ± 10.42 b	282.83 ± 8.21 b
Zhongza	Low VPD	51.46 ± 3.64 a	150.59 ± 4.87 a	202.05 ± 4.94 a	296.21 ± 9.27 a	378.95 ± 18.90 a	337.58 ± 6.53 a
	High VPD	38.01 ± 2.03 b	130.75 ± 4.26 b	168.76 ± 5.54 c	285.99 ± 5.66 ab	247.21 ± 19.28 c	266.60 ± 11.63 b
Cultivar		*	*	NS	*	NS	NS
Treatment		*	**	**	NS	**	**
Cultivar × Treatment		NS	NS	*	NS	**	**

Data are given for the abaxial and adaxial sides of the leaves. Leaf integrated values for the whole leaf are also given: SD = SD_*adaxial*_ + SD_*abaxial*_, SA = (SA_*adaxial*_ + SA_*abaxial*_)/2. Data are means ± standard error (n = 5 plants). Different letters within the same column denote statistically significant differences (Duncan’s test, P < 0.05). Two-way ANOVA was used to estimate the effect of cultivar, treatment, and their interaction (*P < 0.05; **P < 0.01; NS, not significant).

### Plant Hydraulic Conductance

**FIGURE 2:**
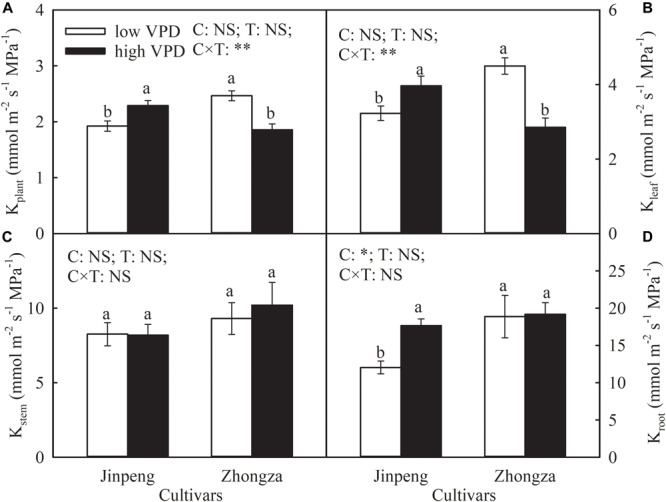
Hydraulic conductance of whole plant (K_plant_) **(A)**, leaf (K_leaf_) **(B)**, stem (K_stem_) **(C)**, and root (K_root_) **(D)** for two tomato cultivars, Jinpeng and Zhongza, grown under low (1.1–1.5 kPa) and high (2.2–2.6 kPa) VPD. Data are means ± standard error (*n* = 5 plants). Different letters denote statistically significant differences (Duncan’s test, *P* < 0.05). Two-way ANOVA was used to estimate the effect of cultivar (C), treatment (T), and their interaction (C × T) (**P < 0.01; *P < 0.05; NS, not significant).

Compared to low VPD, K_plant_ and K_leaf_ in Jinpeng significantly increased but in Zhongza declined by 24 and 36%, respectively, under high VPD (Figure 2). No difference in K_stem_ was noted between low and high VPD conditions for either cultivar. K_root_ increased by 46% in Jingpeng under high VPD but was similar under low and high VPD in Zhongza.

**FIGURE 3:**
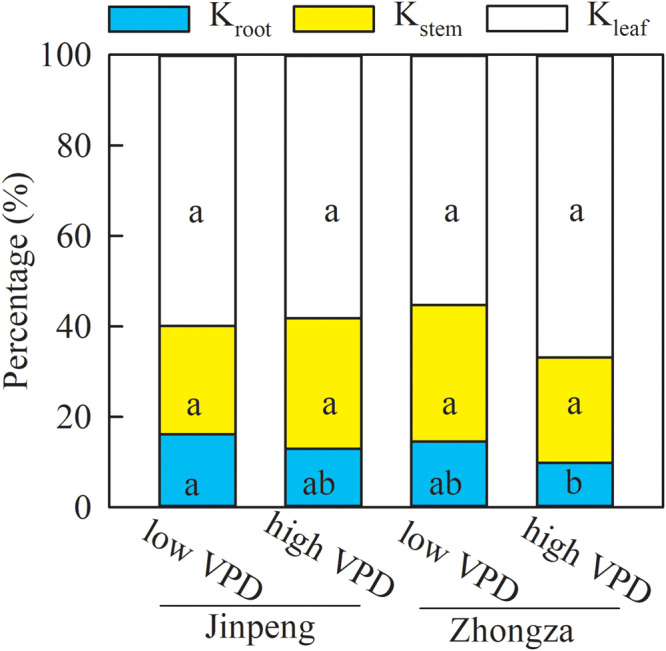
Percentage contributions of leaf (K_leaf_), stem (K_stem_), and root (K_root_) hydraulic conductance to whole plant hydraulic conductance for two tomato cultivars, Jinpeng and Zhongza, grown under low (1.1–1.5 kPa) and high (2.2–2.6 kPa) VPD. Different letters denote statistically significant differences (Duncan’s test, *P* < 0.05).

Relative contributions were analyzed to discern the role of K_leaf_, K_stem_, and K_root_ in the observed changes in K_plant_ (Figure 3). The percentages of K_leaf_, K_stem_, and K_root_ that made up K_plant_ were similar between low and high VPD in both Jinpeng and Zhongza. K_leaf_ accounted for 56–66% of the changes in K_plant_, followed by K_stem_ (26%) and K_root_ (13%).

### Leaf Vein Traits

**FIGURE 4:**
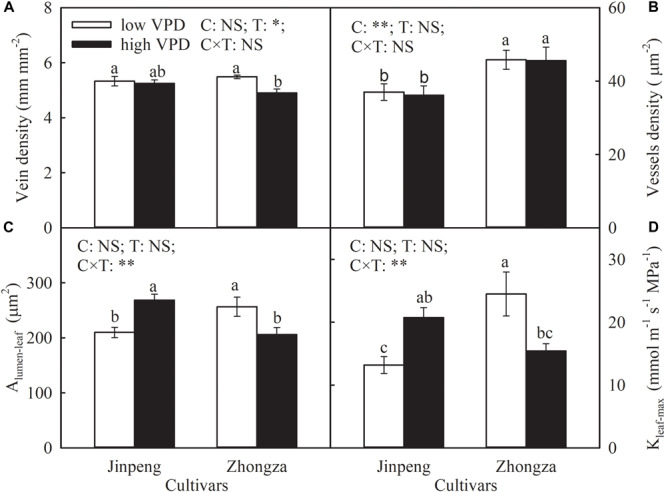
Vein density **(A)**, vessel density **(B)**, lumen area of vessels in leaf vein (A_lumen_-*leaf*) **(C)**, and maximum theoretical leaf vein axial hydraulic conductivity (K_leaf_-*max*) **(D)** for two tomato cultivars, Jinpeng and Zhongza, grown under low (1.1–1.5 kPa) and high (2.2–2.6 kPa) VPD. Data are means ± standard error (*n* = 5 plants). Different letters denote statistically significant differences (Duncan’s test, *P* < 0.05). Two-way ANOVA was used to estimate the effect of cultivar (C), treatment (T), and their interaction (C × T) (^∗∗^*P* < 0.01; ^∗^*P* < 0.05; NS, not significant).

Vein density was not affected by VPD treatment in Jinpeng, but declined in Zhongza under high VPD compared to low VPD (Figure 4). For both cultivars, high VPD had no influence on vessel density in leaf veins. Under high VPD, A_lumen_-*leaf* and K_leaf_-*max* increased by 28 and 57%, respectively, in Jinpeng but decreased by 20 and 37%, respectively, in Zhongza.

### Stem and Root Morphological Characteristics

**FIGURE 5:**
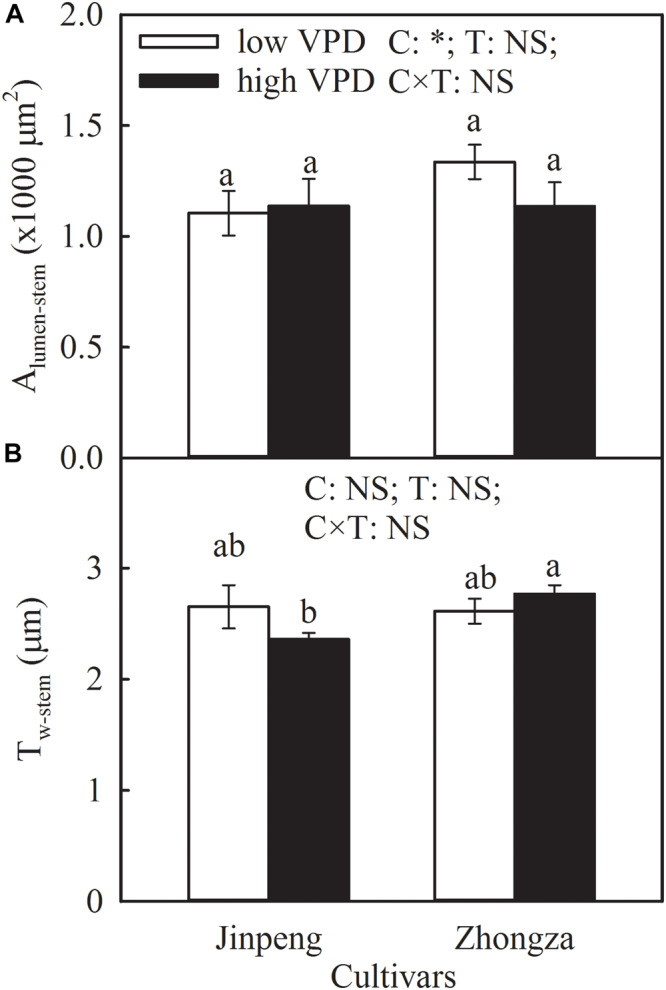
Lumen area (A_lumen_-*stem*) **(A)** and wall thickness (T_w_-*stem*) **(B)** of vessels in stem for two tomato cultivars, Jinpeng and Zhongza, grown under low (1.1–1.5 kPa) and high (2.2–2.6 kPa) VPD. Data are means ± standard error (*n* = 5 plants). Different letters denote statistically significant differences (Duncan’s test, *P* < 0.05). Two-way ANOVA was used to estimate the effect of cultivar (C), treatment (T), and their interaction (C × T) (^∗^*P* < 0.05; NS, not significant).

A_lumen_-*stem* and T_w_-*stem* was not significantly different between low and high VPD in either Jinpeng or Zhongza (Figure 5). Under high VPD, Jinpeng roots had 57, 33, and 17% higher volume, surface area, and average diameter, respectively, than under low VPD (Table 2). No differences in root volume, surface area, or average diameter were found in Zhongza between low and high VPD. Root total length was similar under low and high VPD in both cultivars.

**TABLE 2:** Root volume, surface area, average diameter, and total length for two tomato cultivars, Jinpeng and Zhongza, grown under low (1.1–1.5 kPa) and high (2.2–2.6 kPa) VPD.

**Cultivars**	**Treatments**	**Root volume (cm^3^)**	**Root surface area (cm^2^)**	**Root average diameter (mm)**	**Root total length (cm)**
Jinpeng	Low VPD	0.86 ± 0.08 b	87.44 ± 7.17 b	0.39 ± 0.01 b	708.52 ± 49.62 ab
	High VPD	1.35 ± 0.11 a	116.07 ± 6.50 a	0.46 ± 0.02 a	798.41 ± 50.90 a
Zhongza	Low VPD	1.36 ± 0.14 a	104.25 ± 8.73 ab	0.51 ± 0.03 a	653.36 ± 34.47 b
	High VPD	1.37 ± 0.12 a	103.50 ± 6.99 ab	0.48 ± 0.02 a	721.20 ± 53.97 ab
Cultivar		*	NS	**	NS
Treatment		*	NS	NS	NS
Cultivar × Treatment		NS	NS	*	NS

Data are means ± standard error (n = 5 plants). Different letters within the same column denote statistically significant differences (Duncan’s test, P < 0.05). Two-way ANOVA was used to estimate the effect of cultivar, treatment, and their interaction (*P < 0.05; **P < 0.01; NS, not significant).

### Growth Analyses

Aboveground dry weight, total dry weight and NAR was similar under high and low VPD in Jinpeng, but these parameters were significantly lower under high VPD than low VPD in Zhongza (Table 3). Although no statistical differences in underground dry weight were found between low and high VPD for both cultivars, it increased by 14% in Jinpeng and decreased by 7% in Zhongza under high VPD.

**TABLE 3:** Aboveground dry weight, underground dry weight, total dry weight, and net assimilation rate (NAR) for two tomato cultivars, Jinpeng and Zhongza, grown under low (1.1–1.5 kPa) and high (2.2–2.6 kPa) VPD.

**Cultivars**	**Treatments**	**Aboveground dry weight (g)**	**Underground dry weight (g)**	**Total dry weight (g)**	**NAR (g m^–2^ d^–1^)**
Jinpeng	Low VPD	1.577 ± 0.079 a	0.119 ± 0.008 a	1.696 ± 0.084 a	2.580 ± 0.147 a
	High VPD	1.482 ± 0.058 a	0.135 ± 0.009 a	1.617 ± 0.063 a	2.357 ± 0.059 ab
Zhongza	Low VPD	1.451 ± 0.105 a	0.123 ± 0.014 a	1.574 ± 0.119 a	2.253 ± 0.078 b
	High VPD	1.177 ± 0.086 b	0.115 ± 0.021 a	1.292 ± 0.106 b	1.847 ± 0.093 c
Cultivar		*	NS	*	**
Treatment		*	NS	NS	**
Cultivar × Treatment		NS	NS	NS	NS

Data are means ± standard error (n = 5 plants). Different letters within the same column denote statistically significant differences (Duncan’s test, P < 0.05). Two-way ANOVA was used to estimate the effect of cultivar, treatment, and their interaction (*P < 0.05; **P < 0.01; NS, not significant).

## Discussion

In the soil–plant–atmosphere continuum, VPD is the driving force for water flow. High VPD induced a higher E in Jinpeng than low VPD, but E was not affected by VPD treatment in Zhongza, suggesting cultivar differences in water dynamic responses to long-term high VPD (Figure 1). Acclimation of water transport pathways for liquid and vapor phases to high VPD may separately or simultaneously occur in plants, leading to a new homeostatic state (Brodribb and Holbrook, 2006; Domec et al., 2009; Simonin et al., 2015; Fernandes-Silva et al., 2016). In the vapor phase, evaporation from the mesophyll surface to the substomatal cavity is enhanced with increasing VPD. The reduced g_s_ in Zhongza under high VPD prevents excessive vapor diffusion out of the leaf while E is proportional to changes in VPD due to unchanged g_s_ in Jinpeng. Additionally, water supply determines how much water can evaporate from the plant. For liquid flow moving through the plant, water supply depends on the K_plant_ for given soil moisture conditions. In Jinpeng, the increased E under high VPD would been maintained by a high K_plant_ (Supplementary Figure S1). The coupling between K_plant_ and E enables leaves to minimize variation in plant water status (Simonin et al., 2015). However, the coordination between K_plant_ and E is disrupted in Zhongza. The reduced K_plant_ suggests a limited water supply in Zhongza under high VPD. Thus, E for Zhongza growing under high VPD is kept at the same level as for low VPD conditions to maintain the balance between water supply and loss.

g_s_ is tightly linked to plant water status (Bunce, 2006; Ripullone et al., 2007). According to the hydraulic feedback hypothesis, a hydraulic feedback loop can describe the mechanism driving the relationship between g_s_ and K_plant_ (Buckley, 2005; Brodribb and Jordan, 2011; Savvides et al., 2012; Simonin et al., 2015). The absence of change in g_s_ and the increase in K_plant_ demonstrated by Jinpeng growing under high VPD suggest that adequate water supply prevents leaf dehydration and therefore the stomata remain open. This acclimation would maximize carbon acquisition under high VPD, which was confirmed by the similar plant biomass and NAR under high and low VPD in Jinpeng (Table 3). In contrast, a coordinated declines in K_plant_ and g_s_ were noted in Zhongza under high VPD (Supplementary Figure S2). Low water transport efficiency has been regarded as the initial cause of water stress symptoms (Fanourakis et al., 2012). Plants are prone to close stomata due to reductions in leaf turgor when subjected to water stress (Martins et al., 2016; Rodriguez-Dominguez et al., 2016). Thus, g_s_ in Zhongza decreases under high VPD to minimize water loss in plants despite limiting carbon acquisition (Table 3). However, the coordination between g_s_ and K_plant_ would also play a role in maintaining the integrity of xylem water transport and reducing the risk of hydraulic failure (Galmés et al., 2013; Liu et al., 2015; Salmon et al., 2015; Du et al., 2018).

Alternatively, the responses of g_s_ to long-term high VPD could be driven by changes in stomatal size and density (Fanourakis et al., 2013; Lu et al., 2015). Franks and Beerling (2009) showed mathematically that g_s_ is positively related to stomatal density and size based on the physics of diffusion through pores. Stomatal size and density decreased in Zhongza under high VPD but no active acclimation to high VPD occurred in Jinpeng. Thus, the reductions in stomatal density and size under high VPD are at least partially responsible for the decline in g_s_ (Table 1 and Supplementary Figure S2). This adjustment in stomatal morphology helps plants avoid the risk of excessive water loss under conditions of high evaporative demand. Furthermore, morphological differences in stomata were more evident in the abaxial epidermis for Zhongza. Fanourakis et al. (2015a) also confirmed that operating g_s_ was mostly situated on the abaxial surface. For plants, the regulation of g_s_ is more effective by changing abaxial stomatal morphology because a broader distribution of stomata in abaxial epidermis.

Due to hydraulic segmentation in the plant water transport system, the hydraulic resistance of the whole plant is partitioned into its functional components related to leaves, stems, and roots (Cruiziat et al., 2002; Sperry and Love, 2015). Our study showed that K_leaf_ determines approximately 60% of the changes in K_plant_ (Figure 3). Moreover, a synchronized increase in K_plant_ and K_leaf_ was found for Jinpeng whereas a coordinated decrease in K_plant_ and K_leaf_ occurred for Zhongza under high VPD (Figure 2). Thus, K_plant_ was mostly dominated by K_leaf_, which is consistent with previous studies even though leaves constitute less than 3% of the pathway for water flow through the whole plant (Sack et al., 2003; Domec et al., 2009; Tabassum et al., 2016).

During water movement in the leaf, leaf vein structural characteristics are a substantial constraint on K_leaf_ (Sack and Holbrook, 2006; Carins Murphy et al., 2014; Xiong et al., 2017). The decline in vein density of Zhongza under high VPD means not only a reduction in the surface area for the exchange of xylem water with surrounding tissue but an increase in distance for water movement from the xylem into mesophyll cells (Roth-Nebelsick et al., 2001; Sack and Frole, 2006), therefore resulting in a decrease in K_leaf_. Additionally, A_lumen_-*leaf* also declined in Zhongza under high VPD (Figure 4 and Supplementary Figure S3). A small vessel diameter in general corresponds to a high resistance for water transport, but it can withstand very low negative pressure without generating an embolism (Pittermann and Sperry, 2003). In contrast, Jinpeng showed a unaffected leaf vein density and synchronized increases in A_lumen_-*leaf* and E under high VPD (Figure 4 and Supplementary Figure S3), suggesting that large vessel diameter plays a critical role in improving K_leaf_ and maintaining E.

Interestingly, high VPD treatment also resulted in an increase in K_root_ and a concurrent increase in root volume, surface area, and average diameter for Jinpeng whereas no acclimations of K_root_ and root morphology were observed in Zhongza (Table 2 and Supplementary Figure S4). Meanwhile, the large root was tightly related to high E. Modifications in root morphology would improve hydraulic properties at the soil-root interface, promoting water uptake and transport (Steudle, 2000; Domec et al., 2009). However, this active acclimation in root and leaf veins is likely to occur in plants that can maintain a high carbon acquirement because of a high carbon investment. Plants with closed stomata under high VPD would prefer to reduce the investment to aboveground according to the multiple limitation hypothesis (Farrior et al., 2013; Sellin et al., 2015). This may explain the different changes in plant biomass and NAR between Jinpeng and Zhongza under high VPD (Table 3). In the present study, no changes in K_stem_, A_lumen_-*stem* and T_w_-*stem* were found under high VPD in either cultivar (Figure 5). Thus, the major role in regulating K_plant_ under high VPD is attributed to the leaf and root, as previously reported for several tree species (Domec et al., 2009; Torres-Ruiz et al., 2015).

## Conclusion

The present study indicates that different hydraulic regulation strategies are responsible for the discrepancies found in terms of water dynamics in the cultivars studied. Furthermore, the main results of the present study reinforce the idea that the responses of water dynamics to high VPD depend on acclimation at the whole plant level, particularly at the stomatal, leaf and root levels. High carbon investment in the hydraulic architecture of leaves and roots warrants a sufficient water supply to meet the requirement of water loss, maintaining g_s_. In contrast, plants are prone to triggering a simultaneous decrease in g_s_ and K_leaf_ to prevent excessive water loss when environmental factors have a negative effect on the leaf hydraulic system. Therefore, high VPD treatment would impose a tradeoff between the water and carbon economy of the plant.

## Data Availability Statement

The datasets generated for this study are available on request to the corresponding author.

## Author Contributions

QD, XJ, and JL conceived and designed the experiments. QD, XS, JZ, PB, and JD performed the experiments. QD and XJ analyzed the data and wrote this manuscript. JL contributed extensively to its finalization.

## Conflict of Interest

The authors declare that the research was conducted in the absence of any commercial or financial relationships that could be construed as a potential conflict of interest.

## References

[B1] Aliniaeifard S. Matamoros P. M. van Meeteren U. (2014). Stomatal malfunctioning under low VPD conditions: induced by alterations in stomatal morphology and leaf anatomy or in the ABA signaling? *Physiol. Plant.* 152 688–699. 10.1111/ppl.12216 24773210

[B2] Allen C. D. Breshears D. D. McDowell N. G. (2015). On underestimation of global vulnerability to tree mortality and forest die-off from hotter drought in the Anthropocene. *Ecosphere* 6 129 10.1890/es15-00203.1

[B3] Berlyn G. P. Miksche J. P. (1976). *Botanical Microtechnique and Cytochemistry. The.* Ames, IA: Iowa State University Press.

[B4] Brodribb T. J. Holbrook N. M. (2006). Declining hydraulic efficiency as transpiring leaves desiccate: two types of response. *Plant Cell Environ.* 29 2205–2215. 10.1111/j.1365-3040.2006.01594.x 17081253

[B5] Brodribb T. J. Jordan G. J. (2011). Water supply and demand remain balanced during leaf acclimation of Nothofagus cunninghamii trees. *New Phytol.* 192 437–448. 10.1111/j.1469-8137.2011.03795.x 21679190

[B6] Buckley T. N. (2005). The control of stomata by water balance. *New Phytol.* 168 275–292. 10.1111/j.1469-8137.2005.01543.x 16219068

[B7] Bunce J. A. (2006). How do leaf hydraulics limit stomatal conductance at high water vapour pressure deficits? *Plant Cell Environ.* 29 1644–1650. 10.1111/j.1365-3040.2006.01541.x 16898024

[B8] Carins Murphy M. R. Jordan G. J. Brodribb T. J. (2014). Acclimation to humidity modifies the link between leaf size and the density of veins and stomata. *Plant Cell Environ.* 37 124–131. 10.1111/pce.12136 23682831

[B9] Cruiziat P. Cochard H. Ameglio T. (2002). Hydraulic architecture of trees: main concepts and results. *Ann. For. Sci.* 59 723–752. 10.1051/forest:2002060

[B10] de Boer H. J. Price C. A. Wagner-Cremer F. Dekker S. C. Franks P. J. Veneklaas E. J. (2016). Optimal allocation of leaf epidermal area for gas exchange. *New Phytol.* 210 1219–1228. 10.1111/nph.13929 26991124 PMC5069575

[B11] Dewar R. Mauranen A. Makela A. Holtta T. Medlyn B. Vesala T. (2018). New insights into the covariation of stomatal, mesophyll and hydraulic conductances from optimization models incorporating nonstomatal limitations to photosynthesis. *New Phytol.* 217 571–585. 10.1111/nph.14848 29086921

[B12] Domec J. C. Noormets A. King J. S. Sun G. McNulty S. G. Gavazzi M. J. (2009). Decoupling the influence of leaf and root hydraulic conductances on stomatal conductance and its sensitivity to vapour pressure deficit as soil dries in a drained loblolly pine plantation. *Plant Cell Environ.* 32 980–991. 10.1111/j.1365-3040.2009.01981.x 19344336

[B13] Du Q. J. Liu T. Jiao X. C. Song X. M. Zhang J. Y. Li J. M. (2019). Leaf anatomical adaptations have central roles in photosynthetic acclimation to humidity. *J. Exp. Bot.* 70 4949–4962. 10.1093/jxb/erz238 31145790

[B14] Du Q. J. Xing G. M. Jiao X. C. Song X. M. Li J. M. (2018). Stomatal responses to long-term high vapor pressure deficits mediated most limitation of photosynthesis in tomatoes. *Acta Physiol. Plant.* 40 149 10.1007/s11738-018-2723-7

[B15] Fanourakis D. Carvalho S. M. P. Almeida D. P. F. van Kooten O. van Doorn W. G. Heuvelink E. (2012). Postharvest water relations in cut rose cultivars with contrasting sensitivity to high relative air humidity during growth. *Postharvest Biol. Technol.* 64 64–73. 10.1016/j.postharvbio.2011.09.016

[B16] Fanourakis D. Heuvelink E. Carvalho S. M. P. (2013). A comprehensive analysis of the physiological and anatomical components involved in higher water loss rates after leaf development at high humidity. *J. Plant Physiol.* 170 890–898. 10.1016/j.jplph.2013.01.013 23474196

[B17] Fanourakis D. Giday H. Milla R. Pieruschka R. Kjaer K. H. Bolger M. (2015a). Pore size regulates operating stomatal conductance, while stomatal densities drive the partitioning of conductance between leaf sides. *Ann. Bot.* 115 555–565. 10.1093/aob/mcu247 25538116 PMC4343285

[B18] Fanourakis D. Heuvelink E. Carvalho S. M. P. (2015b). Spatial heterogeneity in stomatal features during leaf elongation: an analysis using Rosa hybrida. *Funct. Plant Biol.* 42 737–745. 10.1071/FP15008 32480717

[B19] Fanourakis D. Hyldgaard B. Giday H. Aulik I. Bouranis D. Körner O. (2019). Stomatal anatomy and closing ability is affected by supplementary light intensity in rose (Rosa hybrida L.). *Hortic. Sci.* 46 81–89. 10.17221/144/2017-HORTSCI

[B20] Farrior C. E. Tilman D. Dybzinski R. Reich P. B. Levin S. A. Pacala S. W. (2013). Resource limitation in a competitive context determines complex plant responses to experimental resource additions. *Ecology* 94 2505–2517. 10.1890/12-1548.1 24400502

[B21] Fernandes-Silva A. A. López-Bernal Á Ferreira T. C. Villalobos F. J. (2016). Leaf water relations and gas exchange response to water deficit of olive (cv. Cobrançosa) in field grown conditions in Portugal. *Plant Soil* 402 191–209. 10.1007/s11104-015-2786-9

[B22] Franks P. J. Beerling D. J. (2009). Maximum leaf conductance driven by CO2 effects on stomatal size and density over geologic time. *Proc. Natl. Acad. Sci. U.S.A.* 106 10343–10347. 10.1073/pnas.0904209106 19506250 PMC2693183

[B23] Freschet G. T. Violle C. Bourget M. Y. Scherer-Lorenzen M. Fort F. (2018). Allocation, morphology, physiology, architecture: the multiple facets of plant above- and below-ground responses to resource stress. *New Phytol.* 219 1338–1352. 10.1111/nph.15225 29856482

[B24] Galmés J. Ochogavía J. M. Gago J. Roldán E. J. Cifre J. Conesa M. À (2013). Leaf responses to drought stress in Mediterranean accessions of *Solanum lycopersicum*: anatomical adaptations in relation to gas exchange parameters. *Plant Cell Environ.* 36 920–935. 10.1111/pce.12022 23057729

[B25] Grossiord C. Sevanto S. Borrego I. Chan A. M. Collins A. D. Dickman L. T. (2017). Tree water dynamics in a drying and warming world. *Plant Cell Environ.* 40 1861–1873. 10.1111/pce.12991 28556263

[B26] Kono Y. Nakata K. Tatsumi J. (1982). Observations of cross veins of the second foliage leaf blade in the rice plant by use of a revised method for clearing leaves. *Jap. J. Crop Sci.* 51 445–454. 10.1626/jcs.51.445

[B27] Liu Y. Y. Song J. Wang M. Li N. Niu C. Y. Hao G. Y. (2015). Coordination of xylem hydraulics and stomatal regulation in keeping the integrity of xylem water transport in shoots of two compound-leaved tree species. *Tree Physiol.* 35 1333–1342. 10.1093/treephys/tpv061 26209618

[B28] Lu N. Nukaya T. Kamimura T. Zhang D. Kurimoto I. Takagaki M. (2015). Control of vapor pressure deficit (VPD) in greenhouse enhanced tomato growth and productivity during the winter season. *Sci. Hortic.* 197 17–23. 10.1016/j.scienta.2015.11.001

[B29] Macková J. Vašková M. Macek P. Hronková M. Schreiber L. Šantrůček J. (2013). Plant response to drought stress simulated by ABA application: changes in chemical composition of cuticular waxes. *Environ. Exp. Bot.* 86 70–75. 10.1016/j.envexpbot.2010.06.005

[B30] Martins S. C. V. McAdam S. A. M. Deans R. M. DaMatta F. M. Brodribb T. J. (2016). Stomatal dynamics are limited by leaf hydraulics in ferns and conifers: results from simultaneous measurements of liquid and vapour fluxes in leaves. *Plant Cell Environ.* 39 694–705. 10.1111/pce.12668 26510650

[B31] Monteith J. Unsworth M. (1990). *Principles of Environmental Physics*, 2nd Edn London: Edward Arnold.

[B32] North G. B. Lynch F. H. Maharaj F. D. R. Phillips C. A. Woodside W. T. (2013). Leaf hydraulic conductance for a tank bromeliad: axial and radial pathways for moving and conserving water. *Front. Plant Sci.* 4:78. 10.3389/fpls.2013.00078 23596446 PMC3622035

[B33] Pittermann J. Sperry J. (2003). Tracheid diameter is the key trait determining the extent of freezing-induced embolism in conifers. *Tree Physiol.* 23 907–914. 10.1093/treephys/23.13.907 14532014

[B34] Richter H. (1997). Water relations of plants in the field: some comments on the measurement of selected parameters. *J. Exp. Bot.* 48 1–7. 10.1093/jxb/48.1.1

[B35] Ripullone F. Guerrieri M. R. Nole A. Magnani F. Borghetti M. (2007). Stomatal conductance and leaf water potential responses to hydraulic conductance variation in Pinus pinaster seedlings. *Trees* 21 371–378. 10.1007/s00468-007-0130-6

[B36] Rodriguez-Dominguez C. M. Buckley T. N. Egea G. de Cires A. Hernandez-Santana V. Martorell S. (2016). Most stomatal closure in woody species under moderate drought can be explained by stomatal responses to leaf turgor. *Plant Cell Environ.* 39 2014–2026. 10.1111/pce.12774 27255698

[B37] Roth-Nebelsick A. Uhl D. Mosbrugger V. Kerp H. (2001). Evolution and function of leaf venation architecture: a review. *Ann. Bot.* 87 553–566. 10.1006/anbo.2001.1391

[B38] Sack L. Cowan P. D. Jaikumar N. Holbrook N. M. (2003). The ‘hydrology’ of leaves: co-ordination of structure and function in temperate woody species. *Plant Cell Environ.* 26 1343–1356. 10.1046/j.0016-8025.2003.01058.x

[B39] Sack L. Frole K. (2006). Leaf structural diversity is related to hydraulic capacity in tropical rain forest trees. *Ecology* 87 483–491. 10.1890/05-0710 16637372

[B40] Sack L. Holbrook N. M. (2006). Leaf hydraulics. *Annu. Rev. Plant Biol.* 57 361–381. 10.1146/annurev.arplant.56.032604.144141 16669766

[B41] Salmon Y. Torres-Ruiz J. M. Poyatos R. Martinez-Vilalta J. Meir P. Cochard H. (2015). Balancing the risks of hydraulic failure and carbon starvation: a twig scale analysis in declining Scots pine. *Plant Cell Environ.* 38 2575–2588. 10.1111/pce.12572 25997464 PMC4989476

[B42] Savvides A. Fanourakis D. van Ieperen W. (2012). Co-ordination of hydraulic and stomatal conductances across light qualities in cucumber leaves. *J. Exp. Bot.* 63 1135–1143. 10.1093/jxb/err348 22121201 PMC3276089

[B43] Sellin A. Rosenvald K. Õunapuu-Pikas E. Tullus A. Ostonen I. Lõhmus K. (2015). Elevated air humidity affects hydraulic traits and tree size but not biomass allocation in young silver birches (*Betula pendula*). *Fronti. Plant Sci.* 6:860. 10.3389/fpls.2015.00860 26528318 PMC4602113

[B44] Shamshiri R. Che Man H. Zakaria A. Van Beveren P. Wan Ismail W. I. Ahmad D. (2016). Membership function model for defining optimality of vapor pressure deficit in closed-field cultivation of tomato. *Acta Hortic.* 1152 281–290. 10.17660/actahortic.2017.1152.38

[B45] Simonin K. A. Burns E. Choat B. Barbour M. M. Dawson T. E. Franks P. J. (2015). Increasing leaf hydraulic conductance with transpiration rate minimizes the water potential drawdown from stem to leaf. *J. Exp. Bot.* 66 1303–1315. 10.1093/jxb/eru481 25547915 PMC4339593

[B46] Sperry J. S. Adler F. R. Campbell G. S. Comstock J. P. (1998). Limitation of plant water use by rhizosphere and xylem conductance: results from a model. *Plant Cell Environ.* 21 347–359. 10.1046/j.1365-3040.1998.00287.x

[B47] Sperry J. S. Love D. M. (2015). What plant hydraulics can tell us about responses to climate-change droughts. *New Phytol.* 207 14–27. 10.1111/nph.13354 25773898

[B48] Steudle E. (2000). Water uptake by plant roots: an integration of views. *Plant Soil* 226 45–56. 10.1023/a:1026439226716

[B49] Tabassum M. A. Zhu G. Hafeez A. Wahid M. A. Shaban M. Li Y. (2016). Influence of leaf vein density and thickness on hydraulic conductance and photosynthesis in rice (*Oryza sativa* L.) *during water stress*. *Sci. Rep.* 6:36894. 10.1038/srep36894 27848980 PMC5111110

[B50] Torres-Ruiz J. M. Diaz-Espejo A. Perez-Martin A. Hernandez-Santana V. (2015). Role of hydraulic and chemical signals in leaves, stems and roots in the stomatal behaviour of olive trees under water stress and recovery conditions. *Tree Physiol.* 35 415–424. 10.1093/treephys/tpu055 25030936

[B51] Xiong D. L. Flexas J. Yu T. T. Peng S. B. Huang J. L. (2017). Leaf anatomy mediates coordination of leaf hydraulic conductance and mesophyll conductance to CO2 in *Oryza*. *New Phytol.* 213 572–583. 10.1111/nph.14186 27653809

[B52] Xu Z. Z. Zhou G. S. (2008). Responses of leaf stomatal density to water status and its relationship with photosynthesis in a grass. *J. Exp. Bot.* 59 3317–3325. 10.1093/jxb/ern185 18648104 PMC2529243

[B53] Zhang D. L. Jiao X. C. Du Q. J. Song X. M. Li J. M. (2018). Reducing the excessive evaporative demand improved photosynthesis capacity at low costs of irrigation via regulating water driving force and moderating plant water stress of two tomato cultivars. *Agric. Water Manag.* 199 22–33. 10.1016/j.agwat.2017.11.014

[B54] Zsögön A. Alves Negrini A. C. Peres L. E. P. Nguyen H. T. Ball M. C. (2015). A mutation that eliminates bundle sheath extensions reduces leaf hydraulic conductance, stomatal conductance and assimilation rates in tomato (*Solanum lycopersicum*). *New Phytol.* 205 618–626. 10.1111/nph.13084 25267094

