# Supplementary material for: The Response of Water Dynamics to Long-Term High Vapor Pressure Deficit Is Mediated by Anatomical Adaptations in Plants
Source: Front Plant Sci. 2020 Jun 5;11:758. doi: 10.3389/fpls.2020.00758 (PMC7289962; doi:10.3389/fpls.2020.00758)
Supplement: Supplementary file 1 [file Data_Sheet_1.PDF]

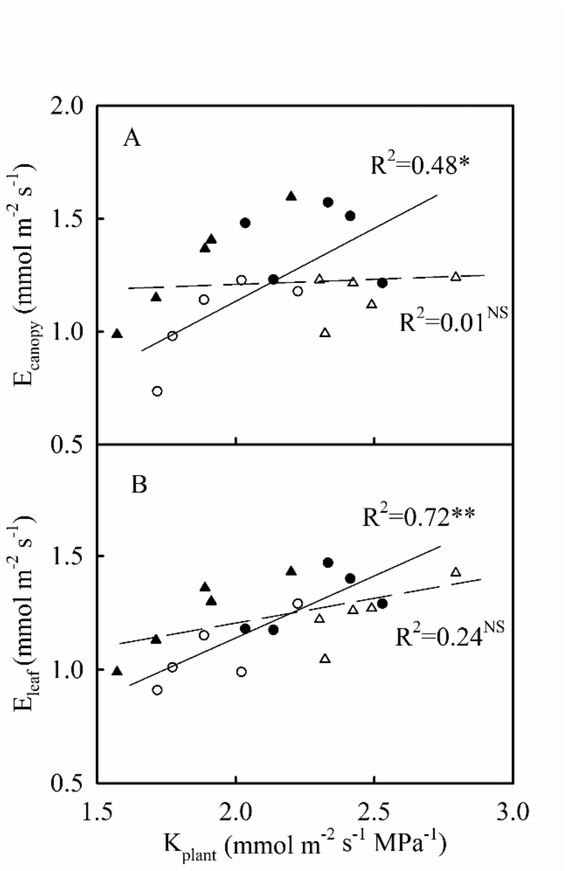

Supplementary Figure S1. Relationship between transpiration rate at the canopy level (E_{canopy}) and hydraulic conductance of whole plant (K_{plant}) (A); Relationship between transpiration rate at the leaf level (E_{leaf}) and K_{plant} (B) for tomato cultivars Jinpeng and Zhongza grown under low (1.1-1.5 kPa) and high (2.2-2.6 kPa) VPD. Open circles, Jinpeng values under low VPD; closed circles, Jinpeng values under high VPD; open triangles, Zhongza values under low VPD; closed triangles, Zhongza values under high VPD. Solid lines represent regressions for Jinpeng, and dashed lines for Zhongza. $^{**}P<0.01$, $^{*}P<0.05$; NS, not significant.

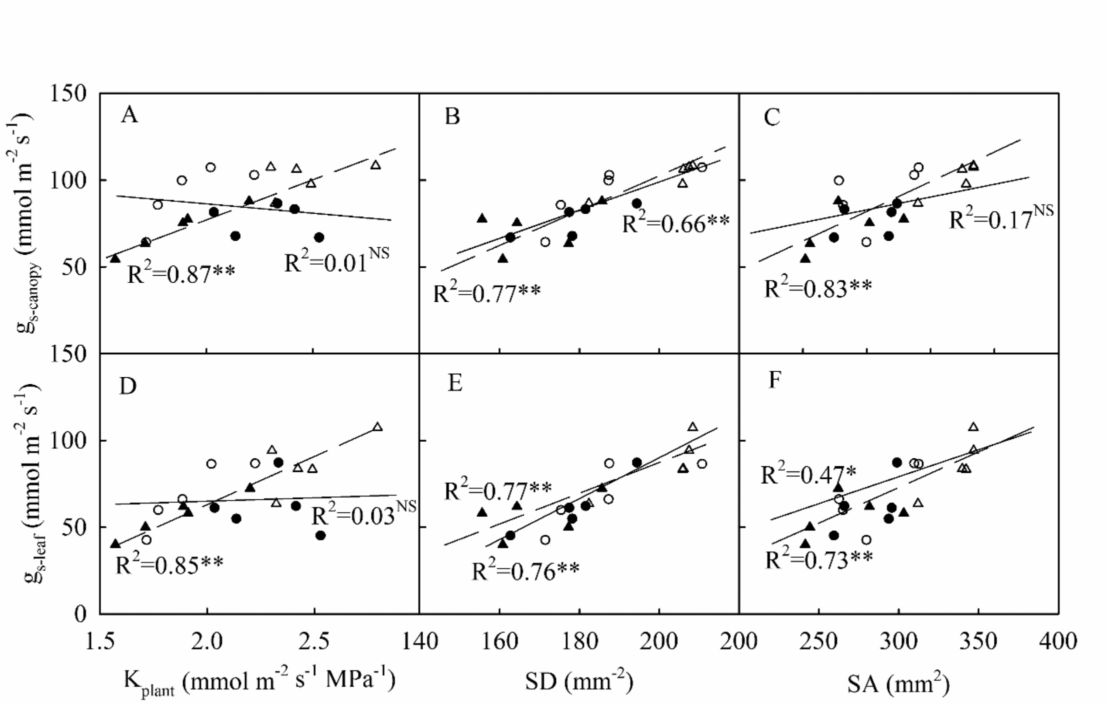

Supplementary Figure S2. Relationship between stomatal conductance at the canopy level ($g_{s\text{-canopy}}$) and hydraulic conductance of whole plant (K_{plant}) (A), stomatal density (SD) (B), and stomatal area (SA) (C); Relationship between stomatal conductance at the and leaf level ($g_{s\text{-leaf}}$) and K_{plant} (D), SD (E), and SA (F) for tomato cultivars Jinpeng and Zhongza grown under low (1.1-1.5 kPa) and high (2.2-2.6 kPa) VPD. Open circles, Jinpeng values under low VPD; closed circles, Jinpeng values under high VPD; open triangles, Zhongza values under low VPD; closed triangles, Zhongza values under high VPD. Solid lines represent regressions for Jinpeng, and dashed lines for Zhongza. **P<0.01, *P<0.05; NS, not significant.

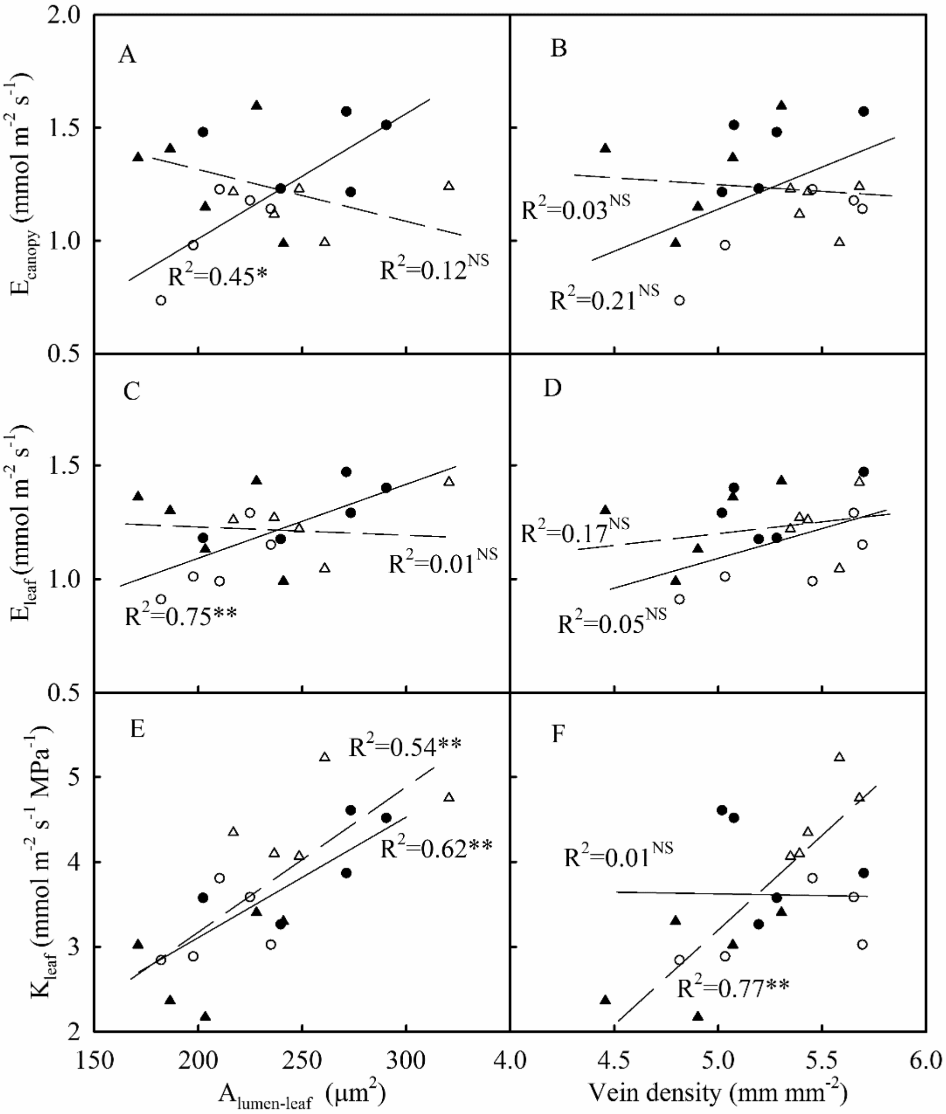

Supplementary Figure S3. Relationship between transpiration rate at the canopy level (E_{canopy}) and lumen area of vessels in leaf vein ($A_{lumen-leaf}$) (A), and vein density (B); Relationship between transpiration rate at the leaf level (E_{leaf}) and $A_{lumen-leaf}$ (C), and vein density (D); Relationship between leaf hydraulic conductance and $A_{lumen-leaf}$ (E), and vein density (F) for tomato cultivars Jinpeng and Zhongza grown under low (1.1-1.5 kPa) and high (2.2-2.6 kPa) VPD. Open circles, Jinpeng values under low VPD; closed circles, Jinpeng values under high VPD; open triangles, Zhongza values under low VPD; closed triangles, Zhongza values under high VPD. Solid lines represent regressions for Jinpeng, and dashed lines for Zhongza. **P<0.01, *P<0.05; NS, not significant.

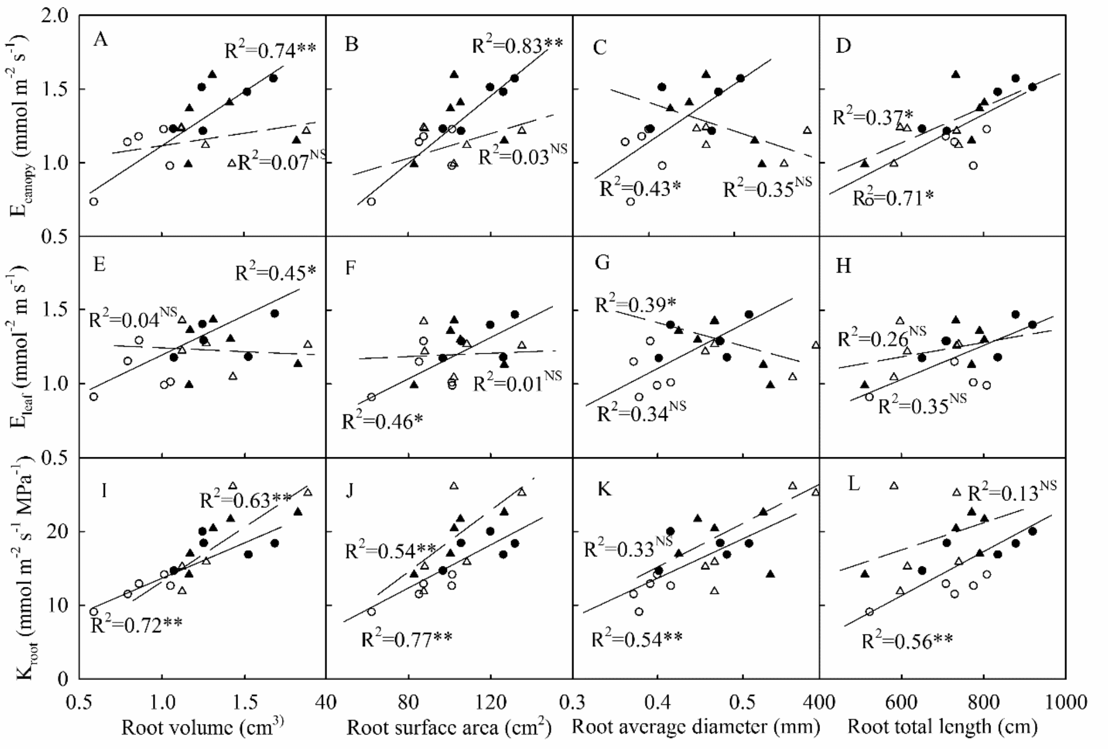

Supplementary Figure S4. Relationship between transpiration rate at the canopy level (E_{canopy}) and root volume (A), root surface area (B), root average diameter (C), and root total length (D); Relationship between transpiration rate at the leaf level (E_{leaf}) and root volume (E), root surface area (F), root average diameter (G), and root total length (H); Relationship between root hydraulic conductance and root volume (I), root surface area (J), root average diameter (K), and root total length (L) for tomato cultivars Jinpeng and Zhongza grown under low (1.1-1.5 kPa) and high (2.2-2.6 kPa) VPD. Open circles, Jinpeng values under low VPD; closed circles, Jinpeng values under high VPD; open triangles, Zhongza values under low VPD; closed triangles, Zhongza values under high VPD. Solid lines represent regressions for Jinpeng, and dashed lines for Zhongza. **P<0.01, *P<0.05; NS, not significant.
